# Interments in the City and Liberties of Philadelphia, from the 24th of July, to the 31st

**Published:** 1841-08-07

**Authors:** 


### HEALTH OF THE CITY

INTERMENTS *in the City and Liberties of Philadelphia, from the 24th of July, to the 31st.*

| Diseases. | Adults. | Children. | Diseases. | Adults. | Children. |
|---|---|---|---|---|---|
| Apoplexy, | 2 | 0 | *Brought forward,* | 39 | 65 |
| Cancer of the womb, | 1 | 0 | Marasmus, | 0 | 6 |
| | | | Malformation, | 0 | 1 |
| Casualty, | 0 | 1 | Mortification, | 1 | 1 |
| Cancrum Oris, | 0 | 1 | Mania a Potu, | 1 | 0 |
| Croup, | 0 | 1 | Old age, | 2 | 0 |
| Congestion of Lungs, | 0 | 1 | Palsy, | 1 | 0 |
| | | | Rupture of Uterus, | 1 | 0 |
| Consumption of the lungs, | 11 | 5 | Scrofula, | 0 | 1 |
| | | | Small pox, | 1 | 1 |
| Convulsions, | 0 | 10 | Still-born, | 0 | 6 |
| Cachexia, | 1 | 0 | Summer Complaint, | 0 | 39 |
| Diarrhœa, | 1 | 8 | | | |
| Dropsy, | 2 | 0 | Suffocation, | 1 | 0 |
| Dropsy, Abdominal, | 1 | 0 | Teething, | 0 | 1 |
| | | | Ulcers, | 1 | 0 |
| —— Head, | 0 | 9 | Unknown, | 3 | 0 |
| Disease of Brain, | 0 | 3 | Worms, | 0 | 1 |
| —— Heart, | 1 | 0 | | — | — |
| Drowned, | 3 | 2 | Total, 172— | 51 | 121 |
| Dysentery, | 5 | 5 | | | |
| Debility, | 0 | 6 | | | |
| Effusion on brain, | 0 | 1 | | | |
| Effects of Laudanum, | 1 | 2 | | | |
| Fever, | 0 | 1 | | | |
| —— Congestive, | 1 | 0 | | | |
| —— Bilious, | 1 | 1 | | | |
| —— Typhus, | 1 | 0 | | | |
| —— Typhoid, | 2 | 0 | | | |
| Inflammation of the Brain, | 2 | 2 | | | |
| —— Throat, | 0 | 1 | | | |
| —— Lungs, | 0 | 2 | | | |
| —— Bowels, | 1 | 2 | | | |
| —— Umbilical, | 0 | 1 | | | |
| Intemperance, | 1 | 0 | | | |
| Jaundice, | 1 | 0 | | | |
| | — | — | | | |
| *Carried forward,* | 39 | 65 | | | |

Of the above, there were

| | |
|---|---|
| under 1 year, | 75 |
| From 1 to 2 | 28 |
| 2 to 5 | 12 |
| 5 to 10 | 1 |
| 10 to 15 | 2 |
| 15 to 20 | 3 |
| 20 to 30 | 13 |
| 30 to 40 | 9 |
| 40 to 50 | 10 |
| 50 to 60 | 7 |
| 60 to 70 | 7 |
| 70 to 80 | 3 |
| 80 to 90 | 2 |
| 90 to 100 | 0 |
| | —— |
| Total, | 172 |

Of the above there were 9 from the almshouse, 19 people of colour, and 1 from the country, which are included in the total amount.

